# Caregiver, community health worker, and dentist feedback on a behavioral intervention for caregivers of children with severe early childhood caries

**DOI:** 10.3389/fpubh.2024.1434475

**Published:** 2024-10-03

**Authors:** Paige Patano, Teresa G. Borowski, Melanie Izquierdo, Calvin Wong, David Avenetti, Helen H. Lee, Joanna Buscemi

**Affiliations:** ^1^Department of Psychology, DePaul University, Chicago, IL, United States; ^2^Institute for Health Research and Policy, University of Illinois Chicago, Chicago, IL, United States; ^3^College of Medicine, University of Illinois Chicago, Chicago, IL, United States; ^4^Department of Anesthesiology, University of Illinois Chicago, Chicago, IL, United States; ^5^Department of Pediatric Dentistry, University of Illinois Chicago, Chicago, IL, United States

**Keywords:** severe early childhood caries, community health worker, behavioral intervention, prevention, parenting

## Abstract

**Introduction:**

Severe early childhood caries (S-ECC) is a common disease within marginalized pediatric populations. S-ECC is often treated under general anesthesia to facilitate extensive treatment in young children, but treatment does not address etiology of an infectious disease that is rooted in health behaviors. Without behavior changes related to toothbrushing and sugar consumption, many children experience recurrent disease and some require subsequent surgeries. To improve post-surgery oral health, we developed PROTECT (Preventing Recurrent Operations Targeting Early Childhood Caries Treatment), a community health worker (CHW)-delivered behavioral intervention for caregivers that focuses on children’s oral health behaviors. The purpose of this study was to use qualitative research methods to receive feedback on the planned protocol for a pilot study of PROTECT, a six-month intervention initiated at the time of a child’s surgery to treat severe early childhood caries.

**Methods:**

Study participants included caregivers of children presenting for surgery [*n* = 12], CHWs [*n* = 8] and dentists [*n* = 8] in a series of audio-video recorded semi-structured interviews. Five coders used Braun and Clarke’s six-phase framework for data analysis.

**Results:**

Participant feedback on the pilot study protocol yielded the following themes: (1) right time, population, and type of support; (2) flexible intervention delivery and content; (3) inclusion of other social determinants of health; and (4) cultural considerations. Implementing a behavioral intervention for caregivers in the immediate time during a child’s surgery for treating dental caries was widely deemed important and timely in order to affect post-surgical behavioral and clinical outcomes. Flexibility in content, timing, and communication were all named as facilitators to participant engagement and study retention. Caregivers and CHWs emphasized the relevance of addressing other social determinants of health. CHWs emphasized the importance of training in becoming aware of culture and practicing with understanding and humility, given the influence on health beliefs, behaviors, and family dynamics. Cultural considerations in intervention delivery were deemed an important factor for participant retention and engagement.

**Discussion:**

Participant feedback led to critical modifications of the pilot study protocol, specifically in intervention content and CHW-led delivery.

## 1 Introduction

Early childhood caries is the most common chronic disease of childhood, globally (1). Young children with severe early childhood caries often require general anesthesia to facilitate extensive treatment. The prevalence of pediatric dental surgery events is common, with treatment of dental caries under general anesthesia serving as the most common reason for surgery among children 1–6 years of age in Canada (2). Unfortunately, because dental surgery under general anesthesia does not address the behavioral etiologic causes of this infectious disease, recurrence of caries is very common (3, 4).

We developed PROTECT (Preventing Recurrent Operations Targeting Early Childhood Caries Treatment), a remotely delivered 6-month behavioral intervention for Medicaid-enrolled caregivers of children presenting for dental surgery under general anesthesia (DGA). Our goal of developing PROTECT was to increase caregiver assisted tooth brushing frequency and reduce added sugar intake among children with early childhood caries presenting for surgery to reduce the risk of subsequent surgeries. We developed a behavioral intervention for caregivers based on our own and others’ previous work on predominant behavior challenges for caregivers whose child present for surgery to treat dental caries (5–12). While there are educational components of the intervention, it is primarily focused on evidence-based behavior change strategies to engage families in problem solving and goal setting around their child’s oral health behaviors. Community health workers (CHWs) will deliver the PROTECT intervention. CHWs have social proximity to our participants and can be trained to address some common social determinants of health for this population (e.g., help with access to medical care, food insecurity, parenting stress) in addition to individual level factors that influence parenting, diet and toothbrushing (13). PROTECT will be delivered over a six-month interval beginning at the surgical event.

In advance of pilot testing interventions, it is necessary to gain feedback from the constituents who will be involved in every stage of research (14). Although many of our PROTECT intervention components (positive parenting, oral health education, behavioral intervention components such as problem solving and goal setting) have been tested in similar populations (15, 16), the combination of (1) intervention content that focuses on evidence-based parenting strategies, (2) identifying health behaviors rather than care as outcomes, and (3) implementation of intervention at the time of a child’s surgery to treat dental caries, when caregivers are processing parental accountability and anxiety for their child, makes PROTECT unique. While there have been other oral health behavior interventions tested in children with caries, to our knowledge, there have not been any interventions that focus on caregiver-child dynamics to change a child’s oral health behaviors. Additionally, we are not aware of any efforts that have tested a behavioral intervention in the immediate period after a child’s surgery for caries treatment. We were concerned about participant engagement, study retention, and unforeseen barriers to our PROTECT intervention. Our study protocol proposed several untested aspects of intervention content and delivery in a marginalized population. Moreover, the study protocol was designed to implement PROTECT during a stressful event for many families, and intervention delivery extended over a six-month period of time. Therefore, prior to finalizing the pilot study protocol for PROTECT, we found it necessary to conduct formative research using qualitative methodology. The purpose of this study was to holistically assess potential barriers to PROTECT regarding both intervention content and planned delivery.

## 2 Methods

### 2.1 Study design and setting

This qualitative study included semi-structured interviews of key constituents (i.e., pediatric dentists, community health workers, and caregivers of children presenting for DGA) and was approved by the Institutional Review Board of the University of Illinois Chicago (UIC). The dental surgeries occurred in an academic dental outpatient setting, adjacent to a university hospital. The academic health organization serves as the primary Medicaid safety net provider for Illinois, particularly for children.

### 2.2 Interview guide development

The semi-structured interview guides were developed through an iterative process and informed by prior work with the surgical population. Interview scripts for all constituents included questions about the planned intervention session topics and delivery (frequency, duration, timing, mode) as well as potential benefits and concerns about the program. Dentists and CHWs were also asked about recruitment, retention, and role of CHWs as interventionists. Dentists were asked how to minimize burden to clinical staff and physicians, and CHWs were asked about social or psychological factors that should be addressed in the PROTECT program. Caregivers were also asked questions about their child’s oral health and dietary behaviors. Spanish versions of the caregiver interview guide and demographic survey were translated, then back translated by bilingual research assistants.

### 2.3 Study population and procedures

The study population consisted of pediatric dentists (*n* = 8), community health workers (CHWs; *n* = 8), and caregivers of young children undergoing DGA (*n* = 12). Data saturation was reached within each group. Current and retired pediatric dentists and CHWs were recruited via email invitations sent to listservs of several organizations. Caregivers received a recruitment flyer at the preoperative clinical visit. On the day of surgery, caregivers were invited to participate in the interview. Informed consent was obtained by the person conducting the interview, which was done in English or Spanish, during the child’s DGA.

Interviews were conducted by three individuals (HL, JB, TB) and one bilingual research assistant. Interviews with pediatric dentists included Zoom interviews (*n* = 5) and an in–person focus group (*n* = 3), conducted between November and December 2022. Interviews with CHWs (*n* = 8) were conducted over Zoom between November 2022 and January 2023. Interviews with caregivers were conducted in January 2023. To reflect the surgical population, 9 English-speaking and 3 Spanish-speaking families were interviewed in-person during the child’s DGA. Two eligible families declined participation. Criteria for recruitment included: English or Spanish-speaking caregivers (aged 18–90 years) of child patients (≤71 months of age) scheduled for DGA at the institution. Exclusion criteria included caregivers of foster children, children with systemic health issues (as classified by the American Society of Anesthesiology classification >3), or children who were not in the same household as the caregiver ≥50% of the week. Participants were also asked to complete a short demographic questionnaire prior to the interview. Interviews lasted 20 to 60 min and were audio recorded. Upon completion of the interview, participants were sent a $50 electronic gift card to compensate them for their time.

### 2.4 Data transcription, translation, coding, and analysis

Interviews were transcribed verbatim using a closed caption software, and transcriptions were reviewed by a research assistant. Spanish interviews were initially transcribed in Spanish and then translated into English by a bilingual research assistant who conducted the interview.

Braun and Clarke’s (2006) six-phase framework (17) was used to analyze this data using Dedoose software (18). Authors (PP, TB, MI, CW, HL) initially went through all interview transcripts and compiled a list of potential codes. Author TB compiled all potential codes, which were then analyzed by author PP to develop an initial codebook. This was then shared with authors (PP, TB, MI, CW, HL) for editing and was continuously edited throughout the coding process as new codes emerged. After developing an initial codebook, two coders were assigned to analyze each interview transcript and add applicable codes to excerpts in the interviews. Authors (PP, TB, and HL) adjudicated any excerpts with divergent coding. After completion of coding and adjudication, consensus of all coders was gained on main themes.

The team consisted of five coders: four identified as female (PP, TB, MI, HL); one identified as Latina (MI), two identified as Asian American (CW, HL), one as Polish American (TB), and one as White (PP); four identified as first-generation Americans (TB, MI, CW, HL).

## 3 Results

Participants (*N* = 28) included 8 pediatric dentists (5 female), 8 CHWs (7 female), and 12 caregivers (see Table 1 for caregiver demographics). We identified 68 codes and four main themes: (1) the proposed intervention provides appropriate support at the right time for this population; (2) flexibility in intervention delivery is necessary for participant engagement; (3) the protocol should address social determinants of health; and (4) cultural considerations.

**Table 1:** Caregiver demographics.

	Race/ethnicity	Gender	Age
Caregiver 1	Black	Female	54
Caregiver 2	Asian	Male	46
Caregiver 3	Hispanic or Latino	Female	29
Caregiver 4	Hispanic or Latino	Female	43
Caregiver 5	Black	Female	40
Caregiver 6	Hispanic or Latino	Female	33
Caregiver 7	White	Female	31
Caregiver 8	Hispanic or Latino	Female	27
Caregiver 9	Black	Female	25
Caregiver 10	Hispanic or Latino	Male	44
Caregiver 11	Hispanic or Latino	Female	25
Caregiver 12	Hispanic or Latino	Male	37

### 3.1 PROTECT is an appropriate intervention for families whose child is undergoing surgery for treatment of early childhood caries

Dentists, CHWs, and caregivers unanimously found the program to be well suited in terms of addressing the common challenges to behavior change with a largely feasible structure that could be implemented at the time of surgery. Each group identified the intervention content to be important, appropriate, and needed. Several perceived benefits of the intervention were acknowledged, such as building parenting skills, creating healthier habits, and improving oral health for future generations. For example, one caregiver said the program was “going to be building important habits with children and with those parents that they could take with them for the rest of their lives,” (Caregiver 9). Similarly, a CHW commented on the importance of the educational content delivered in the intervention, stating that “a lot of these parents…they are young and they are just learning. So, you are giving them information that makes them feel more comfortable in a future setting. Like, ‘Oh, I know about this because I’ve talked about this before.’ So, when they are in a doctor’s office, or anything like that, they feel more prepared.” (CHW 5). One dentist commented “Hopefully…with intervention they are super excited to come in, but a lot of our patients…they think after the surgery like ‘that’s it’ you know, like they do not have to follow up or come back. We want to continue to create good habits when it comes to oral hygiene and dietary habits. So I think it’ll be like a win-win for all parties” (Dentist 8).

Many noted that providing consistent information in a palatable, absorbable way to caregivers with the support of a CHW would be beneficial, particularly to correct misconceptions and address current parental behaviors (e.g., using junk food or candy as rewards, having the child be responsible for brushing). Caregivers showed interest in receiving information on all topics (cavities, healthy eating, problem-solving, etc.). Participants also remarked on the beneficial timing of the intervention – parents are more conscious and focused immediately after surgery. Several caregivers inquired about enrolling themselves into PROTECT at the time of their child’s DGA event.

### 3.2 Flexibility allows for a family-centered intervention

Flexibility of our study protocol was identified as an important characteristic for the pilot study. All groups of participants emphasized the need for flexibility in delivery of intervention content, e.g., the ability to switch topic order to address another topic that is more immediately of concern for the participant. CHWs specifically warned against scripted interventions, saying, “the parents… will tell me something, and I’m like, ‘I have to follow my script,’ and they will feel like I’m not listening to them because they are telling me something else.” (CHW 1). Scripted intervention visits and fixed topical order were cited as barriers to CHW-participant trust and engagement; flexibility in content delivery allowed CHWs to acknowledge and address caregivers’ needs in the moment. For this reason, several participants voiced that relationship-building and trust within the CHW-caregiver relationship was important for engagement and retention. One CHW stated that some caregivers will “probably say that they may not have a need…But then after meeting and talking with them, maybe at some point, they are gonna be like, ‘Okay, well, you asked me about this last time…What kind of information you have about that?’” (CHW 3). Caregivers described times when dentists would deliver education in a manner that was not consistent with the individual needs of the family, e.g., education on soda consumption when this was not an issue for a particular child. Standardized approaches to topical content (families have variable challenges with brushing, sources of sugar, and parenting) and delivery were reported to serve as barriers to engagement, by both CHWs and caregivers.

Dentists, CHWs, and caregivers also expressed the need for flexibility with communication, particularly following the caregiver’s preference for text, phone call, email, or a combination of these modes of communication, as well as contact at non-traditional hours (e.g., outside of 9 a.m. to 5 p.m., on weekends). Participants emphasized that this would be the most effective way to reach caregivers who were often overwhelmed with multiple jobs or the duties of taking care of the household. Flexibility was noted as a necessity for engagement and retention and allows for support that is tailored to the needs of each household.

### 3.3 Inclusion of other social determinants of health

Several dentists and every CHW and caregiver identified a family-centered approach (e.g., “meeting them where they are”) as a necessary characteristic to content and delivery. Participants identified that different families face different challenges, such as stress management and mental health, food insecurity, accessing public programs, and the difficulty of finding (pediatric) dental care under Medicaid. Dentists and CHWs noted that these social determinants were important to address in the intervention to provide support to caregivers when applicable. Caregivers and CHWs recommended that enabling CHWs to address social determinants of health topics, if they spontaneously came up during intervention visits, would not only engender trust and engagement, but would impact caregiver’s mental and emotional bandwidth. One CHW commented that “the more these families feel supported, the more they want to engage in your content of the education. So, this is like, if you are helping them not stress about certain…topics that you have there, then they are gonna be more focused on what you are giving them. So, they do not have to think about that…issue” (CHW 3).

### 3.4 Consideration of culture to facilitate intervention delivery

Across interviews with dentists, CHWs, and caregivers, several facets of culture were identified that required consideration in order to effectively deliver intervention. One important part of culture, unanimously identified across participant groups, was the role of multigenerational parenting in influencing a child’s oral health behaviors. Many caregivers expressed this as a challenge because their children were surrounded by multiple parenting styles and rules were difficult to enforce when their child was under a different family member’s care (e.g., grandparents give the children treats even if the parents say no). Additionally, several caregivers expressed that their own parents did not prioritize oral health due to misconceptions and mistrust of the health care system, which was passed down generationally. Thus, exploring family patterns and understanding generational interactions with care is necessary. CHWs voiced the importance of cultural literacy and humility when interacting with families, particularly with our study population, in order to better understand culturally-based needs and respond in appropriate ways that empowered families and supported a sense of autonomy. One CHW clearly stated, “people from these neighborhoods are a part of the change, and they are involved, and they are not to be toyed with. And they are precious, and they deserve respect and dignity, and a choice and a say in the matter,” (CHW 4). CHWs also brought up the necessity of cultural sensitivity training around subjects such as “healthy” eating, as caregivers may feel judged.

### 3.5 Additional themes

Participant groups were identified based on their differing experiences and perspectives on children’s oral health. Reflective of these differences, we compared and contrasted themes that overlapped or were unique by constituent groupings. Dentists’ discussions oftentimes focused on information around cavity development, brushing quality, and nutrition as well as the role of dental care—some suggested PROTECT address the importance of prevention and preventive visits. Community-based dentists acknowledged the role of Medicaid billing and reimbursement as a provider-level barrier to caring for Medicaid enrolled children.

CHWs provided several recommendations around intervention delivery, such as incorporating visuals, easily digestible key points, and knowledge checks to ensure communication was clear and assess understanding in a population with low health literacy. In addition to general considerations provided in terms of session content and timing, training and recruitment, benefits and concerns, and psychosocial factors, 100% of CHWs were vocal about the need for flexibility based on their own experiences with similar populations.

Discussions with caregivers revealed misunderstandings of health information and a need for improving health literacy. For example, caregivers disclosed that children (even as young as 3–6 years old) were responsible for their own toothbrushing routine, yet the American Academy of Pediatrics recommends that caregivers assist with toothbrushing until about 10 years of age. Caregivers also contradicted themselves when discussing juice and sugary snacks (e.g., “they do not have sugary foods or drinks,” and “they have 8 ounces of organic apple juice a day”).

Caregivers also disclosed frustration with the Medicaid delivery system, describing long wait lists, billing problems, being turned away and stigmatized due to Medicaid status, and feeling judged and shamed by providers. These negative experiences led to feelings of hopelessness, powerlessness, and dehumanization. When discussing Medicaid, one caregiver stated “It feels degrading…I guess as long as you have Medicaid, you really do not matter. They’ll, like, dismiss you quickly. It feels kinda like they do not care” (Caregiver 8). Caregivers also discussed the need for child-centered care from dental providers.

## 4 Discussion

The aim of this study was to assess strengths and potential limitations of the PROTECT intervention, from perspectives of caregivers, CHWs, and dentists. Through qualitative analysis of semi-structured interviews, we determined that our protocol for PROTECT would largely be acceptable in terms of implementing at the time of a child’s surgery and focusing on parenting skills, oral health, and dietary strategies. However, interviews revealed several key modifications, relating to flexibility in intervention delivery and content, inclusion of other social determinants of health, and imbuing cultural consideration throughout content and delivery. All modifications, which led to a more family-centered approach, were identified as facilitators to participant engagement, study retention, and perceived acceptability of our study protocol.

Our findings support the need to build flexibility and family-centered approaches into behavioral interventions. This includes assessing and addressing a wide range of social determinants of health (determined by families, not investigators), building trust within the CHW-caregiver relationship, and making note of communication preferences from family to family. Caregivers and experienced CHWs provided their perspectives based on lived experiences and observations from establishing trust-based relationships. Our findings suggest that flexibility in interventions may help CHWs build rapport with caregivers and translate into caregiver comfort in sharing information about their experiences. In turn, this gives CHWs a better understanding of the caregiver’s needs and thus can provide tailored assistance or support. This need for flexibility and individualization has been found in similar behavioral intervention studies (19–21). A study on the feasibility and acceptability of a behavioral intervention for individuals undergoing methadone treatment found that flexibility in structure, format, and delivery was the most salient facilitator for intervention feasibility (19). Participants in this study found that flexibility accommodated their unique life circumstances, and the flexibility of the interventionist made them feel like they were not being judged. This is consistent with what we found in our interviews. Several caregivers highlighted that receiving health information at the dentist’s office often came off as judgmental, and little attention was paid to their unique individual circumstances. However, caregivers seemed more receptive to a CHW-administered intervention that took their needs into account. Additional studies have also found flexibility as one of the most important facilitators for feasibility in behavioral interventions because it promotes engagement as opposed to following a rigid script and timeline in which participants may feel that it does not apply or does not fit into their lives (20, 21).

Another important theme that emerged in our analysis was the importance of cultural consideration when administering the intervention. Participants mentioned that challenges with multigenerational parenting, oral health misconceptions, and mistrust of the healthcare system were all important factors to consider. Adapting interventions to incorporate culture has been associated with more positive outcomes (22–24). In a systematic review on psychosocial and behavioral interventions for individuals with cancer, individuals undergoing culturally adaptive interventions experienced greater quality of life and well-being, and less distress and anxiety than those in control conditions (22). Behavioral interventions spanning several fields showed similar results of increased efficacy when considering culture in interventions (23, 24). In our interviews, participants anticipated higher retention and engagement as a result of cultural consideration, thus potentially influencing better outcomes in their children’s oral health behaviors.

We report novel findings related to facilitators and barriers to behavior change for families with children who require dental surgery under general anesthesia. Existing literature on caries prevention/oral health promotion evaluates the efficacy of school-based toothbrushing and dietary interventions (25, 26). We are aware of one behavioral intervention that focuses on oral health behaviors (brushing, dietary intake, dental attendance) among children who require surgery under general anesthesia for early childhood caries (27). Although similar in study population, this study protocol differs from PROTECT in nearly every other regard, such as the timing of intervention implementation, frequency, and intervention content. However, the presence of other efforts in the same clinical population highlights the potential for a surgical event to serve as a catalyst from disease to health.

Our study had a few noteworthy limitations. First, results of this study may not generalize to all clinical populations. Our sample is predominantly Medicaid-enrolled families with a child who has experienced severe caries and has required intense treatment. Prior work testing a CHW-led oral health prevention program among 0–3 year olds within urban Chicago families yielded negative findings, which might suggest that experience with severe disease could influence motivation to change oral health behaviors (28). Another limitation to the study is that we do not have demographic information on the dental or CHW participants. Third, one of our interview questions: “How would you feel about a program for caregivers like you that begins the day of your child’s surgery …” may have suggested to some participants that the questions were about “A program,” not necessarily PROTECT. Thus, the participants’ responses may not specifically reflect what they feel about the proposed program. Finally, some of the wording in our interview script may have introduced bias, such as “We believe this type of program is important to prevent recurring childhood dental surgery.” Nevertheless, we believe that our participants were genuinely interested and enthusiastic about the content and intended goals of PROTECT.

These qualitative findings directly informed the next iteration of the PROTECT intervention content, schedule, and training protocols. The CHWs administering the intervention were trained to understand the cultural nuances of our study population based on the feedback received in our interviews. We added flexibility to the intervention; we used multiple modes of communication to reach caregivers; and we added a more comprehensive assessment of social determinants of health and prepared a packet of neighborhood-level resources. Overall, our findings identified strengths and barriers to our study protocol, easing the transition from theory to practice as we conduct our pilot study of a novel intervention. Specifically, qualitative methods enabled us to identify the importance of flexibility in content, timing, and communication in intervention delivery. This flexibility also manifests in greater capacity to address a wider range of social determinants of health. We anticipate that this will translate into enhanced participant bandwidth to engage with their CHW and PROTECT as they work to change health behaviors.

## Data Availability

The raw data supporting the conclusions of this article will be made available by the authors, without undue reservation.
