# Supplementary material for: Caregiver, community health worker, and dentist feedback on a behavioral intervention for caregivers of children with severe early childhood caries
Source: Front Public Health. 2024 Oct 3;12:1434475. doi: 10.3389/fpubh.2024.1434475 (PMC11483999; doi:10.3389/fpubh.2024.1434475)
Supplement: Supplementary file 1 [file Table_1.DOCX]

**Caregiver Interview Script**

**Introduction/Topic Overview:**

Thank you for agreeing to participate.

During this interview, I’d like to discuss your thoughts about your child’s surgery and oral health, including tooth brushing and diet. I’d also like to discuss your thoughts on a program for caregivers like you to provide support in the months following your child’s surgery. We believe this type of program is important to prevent recurring childhood dental surgery.

This should feel like a conversation, so please feel free to be as open and honest as you feel comfortable. There are no right or wrong answers here. If there is any topic that makes you uncomfortable, please feel free to skip. You are the expert on your experience and I am here to learn from you.

I will be audio recording this interview so that I can pay full attention to what you are saying and not miss any details. The recording will be used for research purposes only. Please let me know now if you do not agree to being recorded. You may request that the recording stop at any time.

Do you have any questions for me before we get started?

The recording will begin now (start recording).

**Questions:**

*The first set of questions we’d like to ask are related to your child’s oral and dietary health.*

1. Do you brush your child’s teeth?

*If no: What gets in the way?*

*Are there ways you have been able to get through those barriers in the past?*

2. What do you think the role of tooth brushing has on the development of cavities?

3. When you brush your child’s teeth twice/day, what helps you to accomplish that?

4. When you do not brush your child’s teeth twice a day, what gets in the way?

5. What foods do you think increase risk for cavities?

6. How frequently does your child consume sugary drinks or snacks?

7. What are some challenges to avoiding sugar consumption?

*For this next set of questions, we’d like to hear your thoughts about a program called PROTECT, or Preventing Operations Targeting Early Childhood Caries Treatment, that aims to prevent future dental surgeries for your child. Please feel free to honestly share your thoughts – both positive and negative!*

1. How would you feel about a program for caregivers like you that begins the day of your child’s surgery and continues for 6 months following to support you with tooth brushing and dietary strategies for your child to prevent cavity development?

2. Please take a look at the possible topics that a community health worker would discuss with caregivers as part of the program. [*Participants will be shown a list of topics, including social determinants of oral health*]

- Can you rank the topics according to your priorities?
  - Which topics are most important to you?
  - Which topics are least important to you?
- What other topics that we haven’t mentioned are important to you?

3. Please take a look at the potential session content of the PROTECT program. [*Participants will be shown a table that outlines current planned session content*]. Overall, the content focuses on parenting strategies that may help you to increase your child’s tooth brushing and decrease your child’s sugar consumption. What do you think about these topics?

- What topics would you like covered that aren’t on the list?

4. Please take a look at the schedule of in-person and phone meetings over the course of the

6-month PROTECT program. [*Participants will be shown a table that outlines a schedule of possible CHW interactions*]. What do you think about:

- The frequency of meetings
- The amount of time for each of the meetings
- The timing of the meetings (e.g., during in-person visits including the surgery

date)

5. What do you think would be the benefits of a program like PROTECT?

6. What concerns would you have about a program like PROTECT?

7. What is something you wish dentists knew about your life situation / that would help them better support you in the goal of better oral health for your child?

**Closing Statements:**

Thank you for sharing your thoughts about PROTECT. Is there anything else important to you that I may have missed that we should talk about or that you would like to share?

*(Allow participant to fill in any additional thoughts).*

Thank you for participating in this interview!
