# Supplementary material for: Caregiver, community health worker, and dentist feedback on a behavioral intervention for caregivers of children with severe early childhood caries
Source: Front Public Health. 2024 Oct 3;12:1434475. doi: 10.3389/fpubh.2024.1434475 (PMC11483999; doi:10.3389/fpubh.2024.1434475)
Supplement: Supplementary file 2 [file Table_2.DOCX]

**Interview Script for Community Health Worker**

**Introduction/Topic Overview:**

Thank you for agreeing to participate. During this interview, we will talk about an intervention for caregivers of preschool children presenting for dental surgery under general anesthesia. We are asking about your perspective, as an oral community health worker, on children’s oral health.

We want to understand what makes it easy or difficult for caregivers to help with children’s toothbrushing and sugar intake, including their parenting style. We are interested in social issues and sources of stress that may influence toothbrushing and sugar intake for these families. The program, Preventing Recurrent Operations Targeting Early Childhood Caries Treatment (PROTECT), would focus on harnessing evidence-based parenting interventions to increase tooth brushing and decrease sugar consumption. This intervention would be delivered by community health workers like yourselves.

I will be audio recording this interview so that I can pay full attention to what you are saying and not miss any details. The recording will be used for research purposes only. Please let me know now if you do not agree to being recorded. You may request that the recording stop at any time.

Do you have any questions for me before we get started?

The recording will begin now (start recording).

**Questions:**

*We are going to start by discussing your general thoughts regarding the focus of the PROTECT*

*Intervention, which will help parents as they are helping their children to get better at toothbrushing and also consume less sugar. Community health workers (CHWs) will begin working with parents on the day of a child’s dental surgery and will continue to help them change behaviors through 10-11 discussions over a six-month period.*

1. Please take a look at the session content. Overall, the content focuses on parenting strategies that may help caregivers to increase their tooth brushing and decrease their child’s sugar consumption. What do you think about these topics?

- What other topics you think a CHW should cover that are not on this list?
- What are your thoughts about the order of topics? (e.g., topics that should be addressed earlier or later in the intervention)

1. Please take a look at the proposed schedule of in-person and phone meetings over the course of the 6-month PROTECT program. What do you think about:

- The frequency of meetings
- The amount of time for each of the meetings
- Content timing (do topics need more or less time?)
- The timing of the meetings (e.g., during in-person visits including the surgery date)
- The feasibility of phone and in-person meetings for delivering content

1. As you look at the session content and proposed schedule, what are your thoughts on CHWs working within a fixed, structured visit schedule compared to a flexible visit schedule? (e.g. if a family wants to address social or psychological stress rather than toothbrushing for the first three interactions)
   - What have been your experiences in covering topics with families in a flexible vs. fixed manner (if any)?
2. As you think of social or psychological factors (e.g., caregiver stress, household chaos) that influence a household and a parent and child’s behaviors, do you think those factors should be addressed in a program like this?
   - *If yes, then:*

Do you think it makes sense to have entire sessions that do not address oral health directly, but instead focus on social issues in order to help families change health behaviors?

If so, when do you think is best for these social issues to be addressed during a 6-month intervention?

1. PROTECT will be delivered by community health workers who will be trained by research staff. As you look at the proposed schedule and content, how would you feel about delivering a program like PROTECT?

- What kind of training do you think you’d need to deliver this type of intervention?
- What concerns would you have about delivering this type of intervention given your current training?

1. What do you think are the best ways to recruit dental surgical families for this research study?
2. What might be some barriers to recruitment or retention for these families over a 12-month period?
   - How might we address these barriers?
3. What do you think would be the benefits of a program like PROTECT?
4. What concerns would you have about a program like PROTECT?

**Closing Statements:**

Thank you for sharing your thoughts about PROTECT. Is there anything else important to you that I may have missed that we should talk about or that you would like to share?

Thank you for participating in this interview!
