# Supplementary material for: Caregiver, community health worker, and dentist feedback on a behavioral intervention for caregivers of children with severe early childhood caries
Source: Front Public Health. 2024 Oct 3;12:1434475. doi: 10.3389/fpubh.2024.1434475 (PMC11483999; doi:10.3389/fpubh.2024.1434475)
Supplement: Supplementary file 3 [file Table_3.DOCX]

**Focus Group Script for Moderator: Dental Provider Version**

**Participants:** Moderator, focus group assistant, students

**Arrangement:** Circle seating, refreshments

**Welcome:**

Hello everybody, my name is [insert research team member’s name] and this is [insert research

team member’s name]. I will be guiding the discussion today and [insert research team member’s

name] will be assisting me by taking notes and asking some follow-up questions.

**Topic Overview:**

We invited you all here to talk about an intervention for caregivers of preschool children

presenting for dental surgery under general anesthesia. The program, PROTECT, would focus on

harnessing evidence-based parenting interventions to increase tooth brushing and decrease sugar

consumption.

**Ground Rules:**

One person will be speaking at a time, and we ask you to listen quietly as others share their

views. There are no right or wrong answers and we encourage you to respond to each other’s

answers respectfully when you disagree with something that was said.

This focus group is being audio recorded for research purposes. Please let me know now if you

do not agree to being recorded. You may request that the recording stop at any time. We will be

using only first names during this discussion to keep your identities confidential. Because we

will all hear each other’s answers, I want to emphasize how important it is that you do not share

what we say today with anyone else. By participating in this focus group, you all agree not to

share each other’s identities or share what each other said after you leave today.

My role is to guide the discussion and ask our main questions as we go along today and I will

help to make sure everyone gets the chance to speak. We ask that you silence any cell phones

and keep them put away for the next hour. What questions do you all have for me?

Before we start recording, I would like to go around the room and have everyone introduce

themselves with their name.

The recording will begin now.

**Questions:**

*We are going to start by discussing your general thoughts regarding the focus of the PROTECT*

*intervention. This intervention would begin on the day of a child’s dental surgery and continue 6 months following surgery. A follow-up assessment will occur 6 months post-surgery.*

1. How do you feel about a program for caregivers that begins at the time of surgery and continues for 6 months and provides caregivers with support regarding tooth brushing/dietary strategies to prevent caries development?

2. Please take a look at the proposed schedule of in-person and phone meetings over the

course of the 6-month PROTECT program. What do you think about:

- The frequency of meetings
- The amount of time for each of the meetings
- Content timing (do topics need more or less time?)
- The timing of the meetings (e.g., during in-person visits including the surgery date)

3. Please take a look at the session content. Overall, the content focuses on parenting

strategies that may help caregivers to increase their tooth brushing and decrease their

child’s sugar consumption. What do you think about these topics?

- What other topics would you like covered that aren’t on the list?
- If you could develop this intervention, what topics would you focus on?
- What are your thoughts about the order of topics? (e.g., topics that should be addressed earlier or later in the intervention)

4. PROTECT will be delivered by community health workers who will be trained by

research staff. What concerns, if any, do you have about community health workers

delivering this intervention?

- What, if any, are the benefits of having community health workers delivering this intervention?

5. What burden do you think PROTECT would place on clinic staff/physicians?

- What might minimize this burden?

6. What do you think are the best ways to recruit participants for this research study?

- What might be some barriers to recruitment or retention?
- What are some suggestions to minimize these barriers?

7. What do you think would be the benefits of a program like PROTECT?

8. What concerns would you have about PROTECT?

**Closing Statements:**

Thank you all for participating in our focus group on the PROTECT intervention. Have we

missed anything in talking to you today? (*Allow participants to fill in any additional thoughts,*

*focus group assistant asks any follow-up questions they want to*).

Great, I enjoyed speaking with you all and please be reminded to keep everything that was shared today confidential.
